# Dual Strategies for Enriching Electroactive Microorganisms from Anaerobic Digestate: Carbon-Assisted Acclimation and Direct In Situ Enrichment in a Liter-Scale MFC

**DOI:** 10.3390/bioengineering13060624

**Published:** 2026-05-27

**Authors:** Shiue-Lin Li, Po-Chia Chen, Yun-An Chen, Pei-Ling Chen, Ya-Chun Wei, Tung-Yang Wu, Zone-Ke Lin

**Affiliations:** 1 Department of Environmental Science and Engineering, Tunghai University, Taichung 40704, Taiwan; 2 Taiwan Sugar Research Institute (TSRI), Tainan City 701037, Taiwan; a03370@taisugar.com.tw (Y.-A.C.); a01767@taisugar.com.tw (P.-L.C.)

**Keywords:** microbial fuel cell, livestock wastewater, anaerobic digestate, electroactive microorganisms, carbon powder acclimation

## Abstract

A livestock farm in southern Taiwan produces wastewater with high concentrations of nitrogen and organics, which inhibit anaerobic methanogens and limit the efficiency of its biogas system. To enhance energy recovery, this study developed a liter-scale microbial fuel cell (MFC) system aimed at harvesting electricity from livestock wastewater, serving as a supplementary energy recovery pathway alongside the biogas process. According to the five analyses, the chemical oxygen demand (COD) of raw wastewater ranged from 14 to 21 g L^−1^, with acetate concentrations ranging between 40 and 112 mM. Propionate and butyrate were consistently below 32 mM and 18 mM, respectively. Ammonium ranged from 1.1 to 1.7 g-N L^−1^, indicating the wastewater’s high organic load and elevated nitrogen content. Two liter-scale MFCs, ch5 and ch7, were operated for over 70 d. From days 7 to 28, both MFCs employed a fill-and-draw mode, achieving optimal COD removal exceeding 80%. After resolving leakage issues between days 30 and 40, the system was restarted on day 40, yielding 76% (ch5) and 82% (ch7) of COD removal. Continuous operation began on day 59, and both reactors maintained COD removal rates above 80% for most of the subsequent two-week period. The best power outputs for ch5 and ch7 reached 1.11 and 0.82 W m^−3^, respectively. Although both liter-scale reactors achieved COD removal and measurable power output, the most important finding was obtained from the inoculum comparison experiments. After 54 days of acclimating to raw wastewater solids, no significant current was observed. In contrast, digestate solids acclimated with carbon powder for 22 d produced a peak current of 42.5 A m^−3^ at 147 h, with COD removal rates of 67–73% and complete removal of organic acids. The key conclusion of this study is that anaerobic digestate exhibits electroactive microbial potential, whether operated in liter-scale reactors or acclimated with carbon powder. Further investigation into the microbial community structure is warranted to optimize system performance.

## 1. Introduction

Microbial Fuel Cells (MFCs) are bioelectrochemical systems capable of converting the chemical energy stored in organic matter into electrical energy through microbial catalytic activity [1,2]. In this system, organic substrates are metabolized by microorganisms at the anode under anaerobic conditions, releasing electrons and protons. The substrate acts as the electron donor in this process. Electrons are transferred from the anode to the cathode via an external circuit, while protons migrate to the cathode through a proton exchange membrane. A typical cathodic reaction involves the reduction of oxygen to water, where electrons from the external circuit, protons in the solution, and dissolved oxygen react at the cathode surface [2]. This series of redox reactions ultimately generates electricity [3,4]. Numerous studies have demonstrated that a wide range of compounds can serve as substrates for microbial metabolism in microbial fuel cells (MFCs), including carbohydrates, organic acids, alcohols, and complex wastewaters such as domestic wastewater. In addition, diverse electroactive microorganisms have been reported to contribute to current generation in MFCs, with representative genera including *Geobacter* and *Shewanella*. These findings highlight the broad potential of MFCs for both wastewater treatment and energy recovery [5,6,7,8,9,10,11]. Although all of these microorganisms are capable of generating electricity, their substrate preferences differ, leading to varied applications depending on the specific microbial strain used. Recent studies have shown that a wide range of compounds, including carbohydrates, organic acids, alcohols, and complex wastewaters, can serve as substrates for microbial metabolism in MFCs, highlighting their potential for both wastewater treatment and energy recovery [12,13,14,15]. Although each possesses electricity-generating capabilities, their preferred substrates differ, leading to different application scenarios. The acetate-acclimated sludge was collected from a Southern Taiwan livestock farm. Since the livestock wastewater there contains a high concentration of acetate according to analysis, the sludge acclimated with acetate contains a large number of acetate-utilizing microorganisms. Therefore, it is expected to effectively degrade livestock wastewater, which is characterized by high acetate content, and generate electricity.

In previous studies on wastewater treatment, research has often focused on factors such as electrode materials, biofilms, temperature, pH, substrate loading rate, and external resistance [16]. However, fewer studies have compared the electroactive enrichment potential of different inoculum sources under livestock wastewater conditions, particularly when combined with conductive-material-assisted acclimation strategies [17]. In this study, raw livestock wastewater solids and anaerobic digestate were compared as inoculum sources, and carbon-powder-assisted acclimation was evaluated as a strategy to improve electrochemical startup and electricity generation performance. By integrating small-scale electrochemical reactor tests with liter-scale MFC operation, this study provides new insight into how inoculum type and acclimation affect electroactive enrichment under livestock wastewater conditions.

## 2. Materials and Methods

### 2.1. Substitution of Anolyte and Catholyte Formulations in a Dual-Chamber Electrochemical Reactor

In the dual-chamber electrochemical reactor, 195 mL of anolyte was added to the anode chamber (at a ratio of livestock wastewater/S1/S2 = 120/50/430, with the formulation shown in Table 1); 25 mL of catholyte was added to the cathode chamber (formulated as 100 mM NaH_2_PO_4_, 76 mM KCl, and 80 mM NaOH). The reactor was then placed on a stirrer inside a 35 °C incubator, with the stirring speed set to 5. Afterward, argon gas was introduced, and the system was connected to a potentiostat to begin operation.

**Figure 1:**
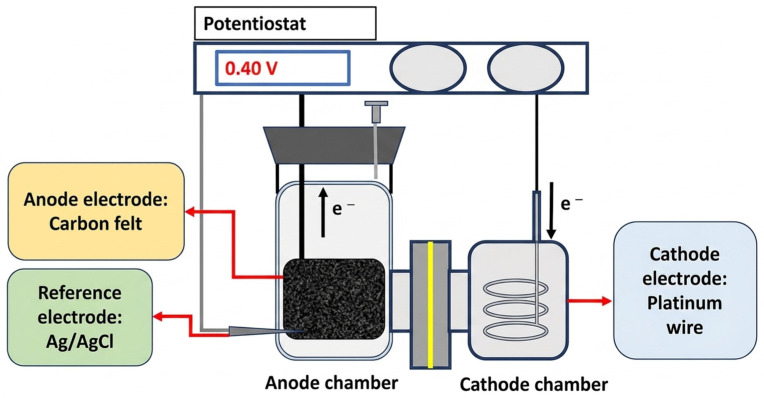
Dual-chamber electrochemical reactor. Red arrows indicate component annotations, whereas black arrows indicate the direction of electron flow.

The dual-chamber electrochemical reactor (Figure 1) consists of an anode chamber and a cathode chamber. In the anode chamber, the graphite felt electrode is secured with a Teflon gasket, and a platinum wire is passed through the titanium plate and the carbon felt. The anode and cathode chambers are separated by a proton exchange membrane and a silicone gasket. After the two chambers are tightened and fixed with aluminum clamps and stainless-steel screws, the reactor is sterilized in an autoclave. Inside a biosafety cabinet, the silver/silver chloride reference electrode wrapped with waterproof tape is inserted into the lower part of the anode chamber, and the silicone cap equipped with a pressure-balance filter head is tightly sealed. The cathode chamber is filled with 25 mL of phosphate-buffered solution. After assembly, the reactor is placed on a stirrer inside a 35 °C incubator with the stirring speed set to 5. The anode, cathode, and reference electrodes are connected to a potentiostat, with the working electrode potential controlled at +0.4 V (vs. Ag/AgCl reference electrode). The data logger (HA-151A, Hokuto Denko, Tokyo, Japan) records the response current, and chronoamperometry is performed during operation. After inoculation, the electrochemical reactor tests were operated for approximately 11 days to monitor startup behavior and current generation.

### 2.2. Electrochemical Analysis Methods

Cyclic voltammetry (CV) is an analytical method used to determine redox reactions occurring at specific voltages and can be applied to identify unknown electron-transfer mediators in samples or analyze changes in electrochemical equilibria. The procedure involves using a potentiostat (CHI611A Electrochemical Analyzer, CH Instruments, Inc., Bee Cave, TX, USA) to linearly scan back and forth between a low potential and a high potential at a scan rate of 1 mV s^−1^. The resulting plot of voltage (*E*) versus current (*I*) is then interpreted. During measurement, three electrodes must be connected: the working electrode (carbon felt), the reference electrode (Ag/AgCl electrode), and the auxiliary electrode (platinum wire). The observed redox peaks correspond to the redox reactions occurring on the working electrode.

### 2.3. Water Quality Analysis Method

High-performance liquid chromatography (HPLC) was used to analyze organic acids and to monitor changes in organic acids during microbial metabolic degradation, including acetate degradation, propionate degradation, and butyrate degradation. The peak areas obtained from chromatograms were applied to calibration curves to calculate the concentrations of different organic acids, and real-time sampling was used to understand microbial metabolic activity. The HPLC system consisted of a CTD-10A pump, an LC-10AD column oven, and an RID-10A UV detector (Shimadzu Corporation, Kyoto, Japan). The mobile phase was a 0.08 M sulfuric acid solution, flowing through an ICSep COREGEL-87H3 column at a rate of 0.5 mL min^−1^. The column oven temperature was set at 40 °C to facilitate the separation of organic acids. Chemical oxygen demand (COD), suspended solids (SS), and ammonia nitrogen were determined following the Standard Method [18].

### 2.4. Assembly and Operation of the Liter-Scale MFC

**Figure 2:**
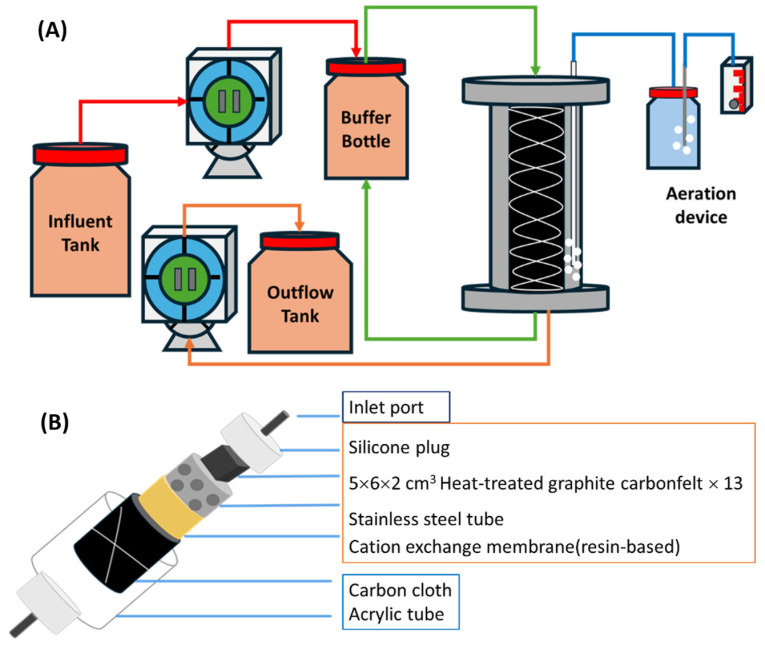
Schematic diagram of the liter-scale MFC. (**A**) Process flow diagram; (**B**) Internal design of the reactor. Red arrows indicate wastewater flow, green arrows indicate internal recirculation flow, and blue arrows indicate gas flow.

The construction of the scaled-up MFC reactor was carried out according to the design shown in Figure 2. The anode chamber was a stainless-steel cylindrical reactor (Customization, Iyi Goang Co., New Taipei City, Taiwan) with dimensions of 4.5 cm in diameter and 30 cm in height; the cathode chamber was an acrylic cylindrical reactor (Customization, Iyi Goang Co., Taiwan) with dimensions of 6.5 cm in diameter and 30 cm in height. The anode electrode consisted of 13 pieces of carbon felt (SG-224K, Osaka Gas Co., Osaka, Japan), each measuring 4 × 4 × 2 cm^3^, and was subjected to heat treatment at 450 °C for 10 min before use; The cathode electrode was a single piece of carbon felt (GF030, CeTech Co., Taichung, Taiwan) with dimensions of 20 × 30 × 0.3 cm^3^. The anode and cathode were separated by a 20 × 30 cm^2^ cation exchange membrane (CEM, FKS-PET-130, Fumatech BWT GmbH, Bietigheim-Bissingen, Germany) to maintain ionic transport balance. The influent and effluent of the reactor were conveyed using a peristaltic pump (Model 7554-90, Cole-Palmer, Vernon Hills, IL, USA) at a flow rate of 18.5 mL min^−1^, paired with peristaltic pump tubing (Masterflex, Cole-Palmer, USA) with an inner diameter of 5 mm and a minimum length of 30 cm. The electrodes were connected to the external circuit using titanium wire (Ti Color Alloy Metals Co., New Taipei City, Taiwan), 40 cm in length and 0.32 mm in diameter. The system was equipped with a 500 mL glass serum bottle as a buffer bottle, a 1 L glass serum bottle as the influent bottle, and a 500 mL plastic bottle as the effluent bottle, which were used for substrate storage, influent feeding, and effluent collection, respectively.

The anode chamber was assembled as follows. Epoxy adhesive (Baozhou Resin, Taichung, Taiwan) was applied to the outer wall of the stainless-steel anode reactor, and a pre-cut cation exchange membrane was attached and secured with rubber bands for 24 h to allow complete curing. A carbon felt base layer was placed at the bottom of the chamber, followed by insertion of heat-treated carbon felt pieces threaded through a 40 cm titanium wire. After assembly, rubber stoppers were installed at both ends to seal the column. For the cathode chamber, a single piece of heat-treated carbon felt was wrapped around the outside of the anode chamber and secured with titanium wires positioned 7, 14, 21, and 28 cm above the bottom. Each titanium wire extended approximately 5 cm from the chamber for external electrical connection. The assembled anode chamber was then placed inside an acrylic cathode reactor, inlet and outlet tubing were connected, approximately 550 mL of catholyte was added, and continuous aeration was supplied using an air pump through silicone tubing.

The system was inoculated with acetate-acclimated sludge as the mixed microbial culture, which was added to the buffer bottle (working volume 300 mL) installed between the peristaltic pump and the MFC reactor. The peristaltic pump delivered the anolyte into the top of the anode chamber and withdrew it from the bottom back into the buffer bottle to form a recirculation loop (flow rate approximately 18.5 mL min^−1^). The total volume of anolyte in the anode chamber and the buffer bottle was 1 L. During operation, 120 mL of anolyte was withdrawn from the buffer bottle each time and replaced with an equal volume of sterile, filtered raw wastewater to supply substrate for the microorganisms, enabling semi-batch operation.

During continuous-flow operation, influent was fed to the system at 5 mL h^−1^ using peristaltic pumps. The effluent was divided into discharge and recirculation streams to maintain internal liquid circulation. A 1 kΩ external resistor was applied throughout operation. Organic acids, COD, and ammonia nitrogen were periodically analyzed, and voltage was continuously recorded. Polarization and power density curves were measured to evaluate electrochemical performance.

This study conducted two continuous-flow operations. In the first run, livestock wastewater was diluted to 1/5 strength to prepare 1 L of anolyte, with the following components added in addition to the wastewater: MgSO_4_·7H_2_O, 1.7 mM; NH_4_Cl, 3.13 mM; NaH_2_PO_4_, 1.79 mM; KH_2_PO_4_, 0.26 mM; K_2_HPO_4_, 1 mM; FeCl_3_, 57.12 nM; H_3_BO_3_, 24.48 nM; CuSO_4_, 1.22 nM; KI, 11.22 nM; MnCl_2_, 6.22 nM; Na_2_MoO_4_, 2.52 nM; ZnSO_4_, 4.25 nM; CoCl_2_, 6.44 nM; NiCl_2_, 0.86 nM. The dual-chamber system was operated for 13 days. In the second run, livestock wastewater was diluted to 3/5 strength to prepare 2 L of substrate solution, with the following components added in addition to the wastewater: MgSO_4_·7H_2_O, 0.85 mM; NH_4_Cl, 1.57 mM; NaH_2_PO_4_, 0.89 mM; KH_2_PO_4_, 0.13 mM; K_2_HPO_4_, 0.5 mM; FeCl_3_, 28.56 nM; H_3_BO_3_, 12.24 nM; CuSO_4_, 0.61 nM; KI, 5.61 nM; MnCl_2_, 3.11 nM; Na_2_MoO_4_, 1.26 nM; ZnSO_4_, 2.13 nM; CoCl_2_, 3.22 nM; NiCl_2_, 0.43 nM. The system was operated for 21 days and 15 days, respectively. During non-continuous-flow periods, the system was operated in semi-batch mode, and reactor maintenance was performed. The long-run operation of the liter-scale MFCs was conducted for 78 days in CH5 and 71 days in CH7, including semi-batch and continuous-flow phases. This study did not include direct analyses of microbial deactivation or poisoning, such as cell viability, toxicity, or inhibition assays.

### 2.5. Power Generation Potential Test of Acclimated Raw Wastewater and Digestate Solids

For the acclimation experiment, a 500 mL serum bottle fitted with a GL-45 perforated screw cap and silicone stopper was used as the acclimation reactor. Raw wastewater solids were prepared by removing coarse foreign materials from the wastewater, followed by repeated centrifugation at 6500 rpm for 5 min to collect the solids. The collected solids were dispersed in phosphate-buffered saline (PBS, pH 7.0) to obtain 50 mL of concentrated solids with a VSS concentration of approximately 22 g L^−1^, which was used as inoculum. Digestate solids were obtained from the solid fraction separated from anaerobic effluent from the Southern Taiwan livestock farm and similarly dispersed in PBS to obtain 50 mL of concentrated solids with a VSS concentration of approximately 35 g L^−1^.

The initial acclimation medium contained NaH_2_PO_4_ (4 mM), KCl (3.2 mM), MgSO_4_ (0.04 mM), NH_4_Cl (1.12 mM), acetate (54 mM), and trace elements including FeCl_3_, H_3_BO_3_, CuSO_4_, KI, MnCl_2_, Na_2_MoO_4_, ZnSO_4_, CoCl_2_, and NiCl_2_. After the addition of the concentrated solids, the total working volume was adjusted to 250 mL. The headspace was flushed with argon before sealing, and the reactor was incubated at 35 °C and 100 rpm in a temperature-controlled orbital shaker. For substrate replacement, the reactor was allowed to settle for 30 min, after which 10 mL of supernatant was withdrawn and replaced with an equal volume of concentrated acclimation substrate. Biogas production was measured daily using a glass syringe to monitor acclimation progress. In addition, following Zhang et al. (2019) [19], 1 g of carbon powder was added to the 250 mL acclimation reactor for carbon-powder acclimation of digestate solids to evaluate its effect on acclimation efficiency. The acclimation experiments for raw wastewater solids and digestate solids were conducted for up to 82 days, depending on the inoculum and treatment condition.

### 2.6. Microbial Community Analysis by Nanopore Sequencing

Microbial community composition was determined by 16S rRNA gene sequencing using the Oxford Nanopore platform. Extracted DNA samples were sent to Source Biotech International/Tri-I Biotech Inc. for sequencing. DNA purity and quality were first checked using a NanoDrop 2000 spectrophotometer (Thermo Scientific, Waltham, MA, USA), and DNA concentration was quantified with a Qubit 2.0 fluorometer (Thermo Fisher Scientific, Waltham, MA, USA) using the Promega QuantiFluor dsDNA System. Sequencing was performed on a MinION Mk1 instrument (Oxford Nanopore Technologies, Oxford, UK) with MinKNOW software version 24.11.8. Sequence processing, including basecalling, demultiplexing, and adapter trimming, was conducted using Dorado version 7.6.7. The processed high-quality reads were used for downstream microbial community analysis. The DNA sequence data generated in this study have been deposited in the NCBI database under BioProject accession number PRJNA1456834.

## 3. Results

### 3.1. Investigation of Livestock Wastewater Characteristics and Component Analysis

Raw wastewater was collected from the Southern Taiwan livestock farm on five occasions between October 2023 and May 2024 for water quality analysis (Table 1). Except for SS and VSS, all parameters were determined in the dissolved fraction after filtration. For raw wastewater, coarse solids were removed before filtration, and the filtrate was used as the anolyte substrate. COD ranged from 14 to 21 g L^−1^, and acetate ranged from 40 to 112 mM, with both reaching their highest values in the second sampling (23 January 2024). Propionate and butyrate remained below 32 and 18 mM, respectively, and were not detected in the fourth sampling. In the first sampling, SS and VSS were 16,583 and 13,750 mg L^−1^, respectively, and these solids were used as inoculum for subsequent acclimation and electrochemical reactor experiments. Ammonia nitrogen ranged from 1.1 to 1.7 g-N L^−1^.

### 3.2. Results of the Liter-Scale MFC Operation

**Figure 3:**
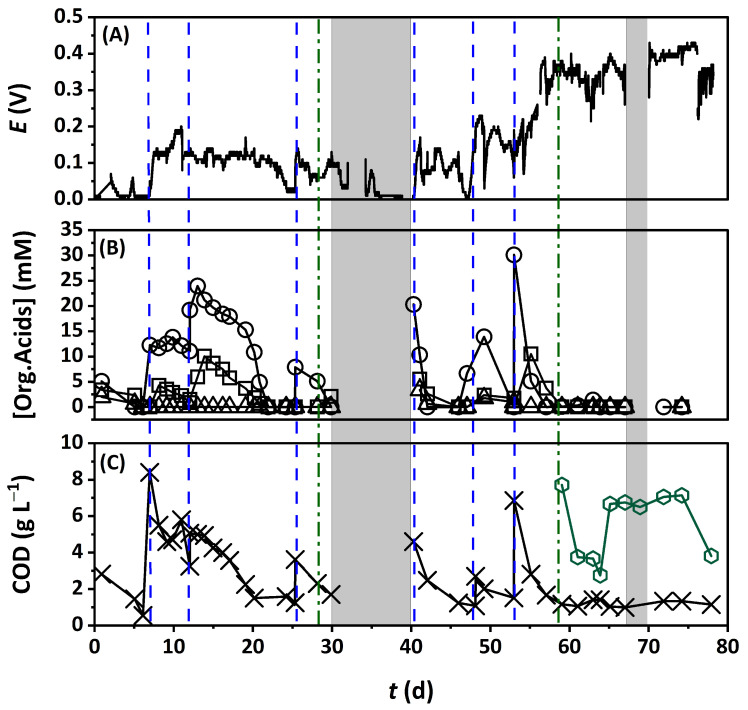
Variation curves of the liter-scale MFC (CH5): (**A**) Voltage, (**B**) Organic acid degradation, (**C**) COD concentration. Open circles: acetate; open squares: propionate; open triangles: butyrate; black ×: COD concentration; open hexagons: influent COD concentration during continuous-flow operation; blue dashed lines: semi-batch substrate feeding times; gray shaded area: leakage period.

The CH5 liter-scale MFC was operated for 78 days (Figure 3). The reactor was run in batch mode until day 7 and then subjected to three Fill-and-Draw feeding events on days 7, 12, and 25, after which continuous-flow operation was initiated on day 28. The initial substrate contained 2.8 g L^−1^ COD, with acetate, propionate, and butyrate concentrations of 5.08, 2.36, and 0.67 mM, respectively. A clear voltage increase was observed after each Fill-and-Draw event. COD removal efficiencies during the three feeding intervals were 80.6%, 40.0%, and 60.8%, respectively. Acetate and propionate were also removed, whereas butyrate was detected only on day 7 and disappeared thereafter. The voltage was maintained at approximately 0.15 V between days 7 and 22, dropped to 0.02 V before the third replenishment, and recovered to 0.1 V after feeding on day 25.

During the first continuous-flow period, the influent consisted of fivefold-diluted raw wastewater collected from the Southern Taiwan livestock farm in March 2024, with a COD concentration of 3054.4 mg L^−1^. Under a hydraulic retention time of 70 h and a COD loading rate of 1.05 kg-COD m^−3^ d^−1^, the voltage gradually increased from 0.06 to 0.11 V after the switch to continuous-flow operation. However, treatment performance remained limited, as the effluent on day 30 still contained 1700 mg-COD L^−1^, although organic acids were not detected. Continuous-flow operation was terminated on day 30 due to reactor leakage.

After day 31, continuous-flow operation was stopped due to leakage, and maintenance was performed. After 40 days, semi-batch operation was resumed, with a total of three feedings on days 40, 47, and 52. After substrate replenishment on day 40, the initial COD concentration was 4.6 g L^−1^, the acetate concentration was 20.3 mM, propionate was 5.5 mM, and butyrate was 3.4 mM. The COD removal efficiencies for each feeding period were 76%, 44%, and 75%, with removal rates of 500, 230, and 1030 mg L^−1^ d^−1^, respectively. For organic acids, acetate removal efficiency was 100% for all feedings, with removal rates of 2.9, 1.3, and 6.0 mM d^−1^. Propionate removal efficiency was also 100% for all feedings, with removal rates of 0.79, 0.46, and 2.09 mM d^−1^. For butyrate, the removal efficiency for the first two feedings was likewise 100%, with removal rates of 0.48 and 0.35 mM d^−1^.

The second continuous-flow operation began on day 59. During the first four days, the COD loading ranged from 0.9 to 2.6 kg-COD m^−3^ d^−1^. After replacing the substrate in the following four days, the loading values became more stable, ranging from 1.3 to 2.2 kg-COD m^−3^ d^−1^. Over the 18-day operation period, fluctuations in the loading values were observed. In terms of COD removal efficiency, values exceeded 80% on all days except days 61 (71%), 62 (62%), 63 (49%), and 77 (70%).

**Figure 4:**
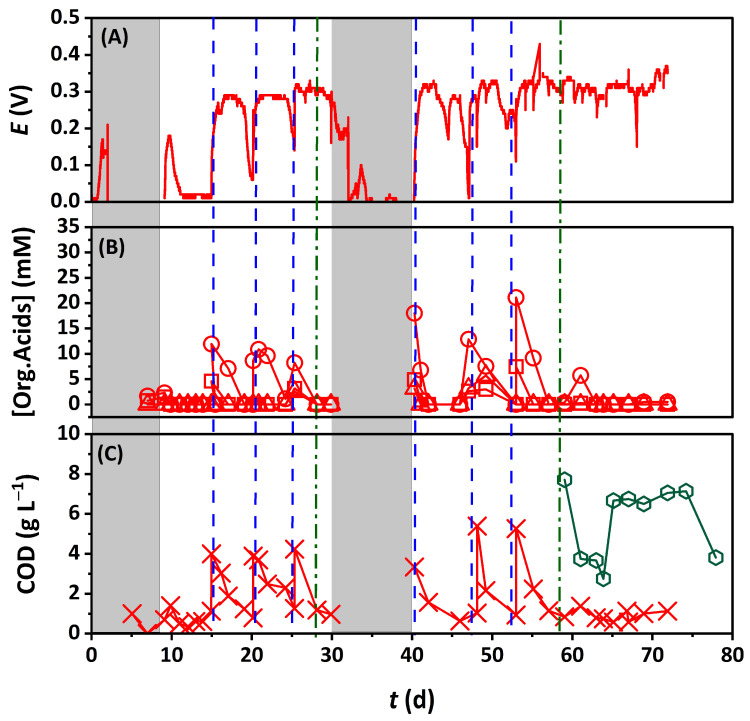
Variation curves of the liter-scale MFC (CH7): (**A**) Voltage, (**B**) Organic acid degradation, (**C**) COD concentration. Open circles: acetate; open squares: propionate; open triangles: butyrate; red ×: COD concentration; open hexagons: influent COD concentration during continuous-flow operation; blue dashed lines: semi-batch substrate feeding times; green dotted lines: continuous-flow influent times; gray shaded area: leakage period.

The CH7 reactor was operated for 71 days (Figure 4). Data from the first 7 days were excluded because the anode chamber was not fully sealed, and leakage occurred. After the repair, Fill-and-Draw batch feeding was initiated. The initial concentrations were 1.4 g-COD L^−1^, 1.65 mM acetate, 0.73 mM propionate, and no detectable butyrate. Although voltage was generated briefly, it soon declined to 0.02 V. Three feeding events were conducted on days 15, 20, and 25 (blue dashed lines). COD removal efficiencies during these periods were 19%, 80%, and 67%, corresponding to removal rates of 54, 640, and 520 mg-COD L^−1^ d^−1^, respectively. Acetate was removed efficiently in all three feeding periods, while propionate was completely removed when detected. Butyrate was not detected throughout the experiment. Following substrate replenishment on day 15, the voltage increased to 0.28 V, and after the second and third feedings, it repeatedly recovered to approximately 0.3 V. Continuous-flow operation began on day 28.

In the first continuous-flow phase, the CH7 reactor was fed with substrate at the same concentration used for CH5. After the switch to continuous flow, the voltage decreased only slightly from 0.30 to 0.26 V. On day 30, organic acids were undetectable in the effluent, and the COD concentration was 967 mg L^−1^, indicating that substrate removal remained effective. Continuous-flow operation was terminated on day 30 because of reactor leakage.

The reactor was restarted on day 40 and operated in semi-batch mode, with feeding events on days 40, 47, and 52. Under semi-batch operation, COD removal efficiencies remained high at 81.2%, 82.6%, and 78.0%, respectively. Acetate and propionate were completely removed in all three feeding periods, and butyrate was completely removed in the first two. The second continuous-flow operation began on day 59 and lasted for 12 days. The influent COD loading and concentration were the same as those used for CH5. In terms of removal efficiency, except for days 61 (63%), 62 (78%), and 63 (73%), all other days achieved efficiencies above 84%. This indicates that the performance of CH7 during the second continuous-flow operation was better than that of CH5.

**Figure 5:**
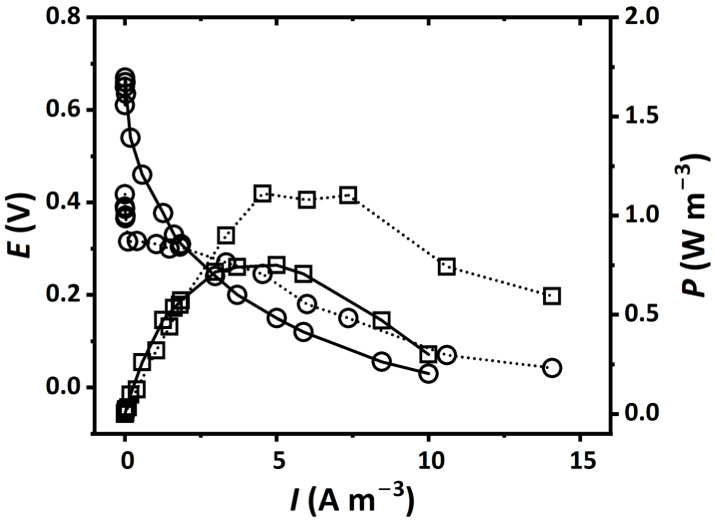
Polarization and power performance of CH5. Circles: voltage; squares: power; solid line: measurement on day 55; dotted line: measurement on day 66.

Figure 5 shows the polarization and power curves of CH5 measured on days 55 and 66. On day 55, the reactor exhibited an OCV of 0.67 V, an internal resistance of 118 Ω, and a maximum power output of 0.75 W m^−3^. By day 66, under continuous-flow operation, the OCV had decreased to 0.40 V, but the internal resistance was reduced to 76 Ω, and the maximum power increased to 1.11 W m^−3^.

**Figure 6:**
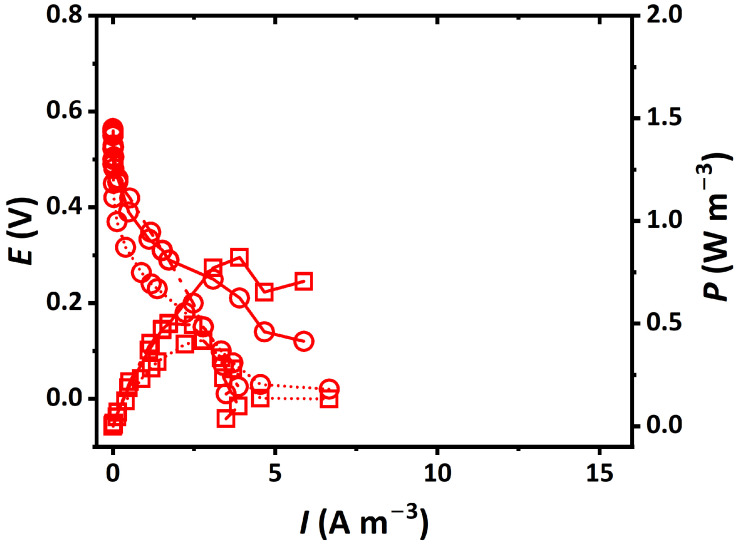
Polarization and power performance of CH7. Circles: voltage; squares: power; solid line: measurement on day 55; dotted line: measurement on day 66; dash-dotted line: measurement on day 70.

Figure 6 shows the polarization and power curves of CH7 measured on days 55, 66, and 70. The OCV remained relatively stable, ranging from 0.50 to 0.54 V. In contrast, internal resistance changed substantially, decreasing from 152.8 Ω on day 55 to 120 Ω on day 66, then increasing sharply to 411 Ω on day 70. The maximum power output was highest on day 55 (0.82 W m^−3^), decreased to 0.42 W m^−3^ on day 66, and partially recovered to 0.5 W m^−3^ on day 70.

Although CH5 and CH7 were operated in parallel, their performances differed markedly. One possible reason is that the larger-scale reactors may have required a longer open-circuit conditioning period to achieve stable startup. This is supported by the observation that CH5 exhibited lower and less stable voltage than CH7 in the early stage, but both reactors converged to approximately 0.30 V after 3 h of open-circuit conditioning before polarization testing on day 55.

Reactor integrity also likely contributed to the discrepancy. CH5 experienced leakage and repair on days 31 and 66, whereas CH7 only showed overflow from the recirculation line on day 66. Such leakage in CH5 may have caused biomass loss and adversely affected performance. Other possible contributing factors include differences in reactor structure, carbon felt distribution, biofilm-induced clogging, inconsistent initial aeration, and insufficient pH control. Taken together, the operation data of CH5 and CH7 indicate that although both reactors were capable of COD removal and electricity generation, their performance still showed noticeable instability during certain periods. These fluctuations were associated with leakage, repair, or operational transition, suggesting that reactor stability remained a significant issue in the present liter-scale system.

### 3.3. Results of Power Generation Potential Tests for Acclimated Raw Wastewater and Digestate Solids

#### 3.3.1. Gas Production and Organic Acid Degradation Results of Wastewater Solids Acclimation with and Without Carbon Powder

**Figure 7:**
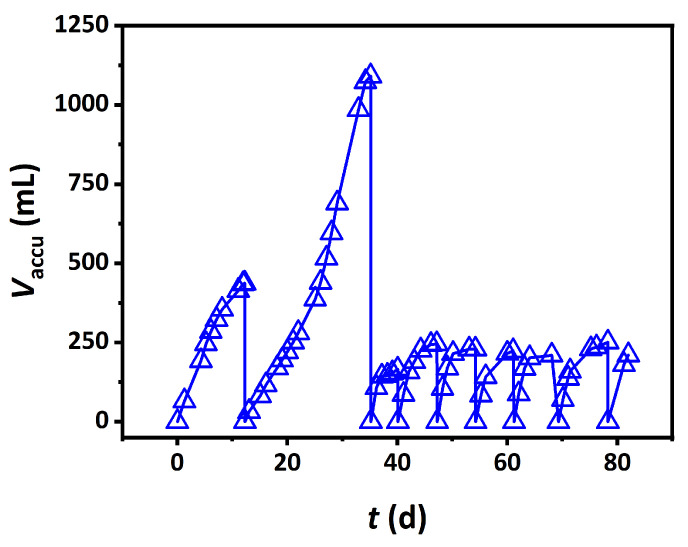
Gas production changes of raw wastewater solids during the acclimation process over time. Open triangles: cumulative gas production for each cycle.

The solid fraction of the raw wastewater collected during the first sampling was acclimated for 82 days (Figure 7). During this period, the substrate was replaced eight times, resulting in nine cumulative gas production profiles. The first two replacement intervals were relatively long (12 and 22 days), whereas the subsequent intervals were approximately 7 ± 2 days. The highest cumulative gas production was observed after the first substrate replacement, reaching 1090 mL (days 12–35). The second-highest cumulative production occurred during the initial period, reaching 437 mL (days 0–12). In the subsequent seven cycles, the average total gas production was approximately 218 mL. After the second substrate replacement, gas production became more stable. The highest short-term gas production among all measurements was observed after the second replacement (days 29–33), reaching 295.2 mL.

#### 3.3.2. Gas Production and Organic Acid Degradation Results of Digestate Solids Acclimation with and Without Carbon Powder

**Figure 8:**
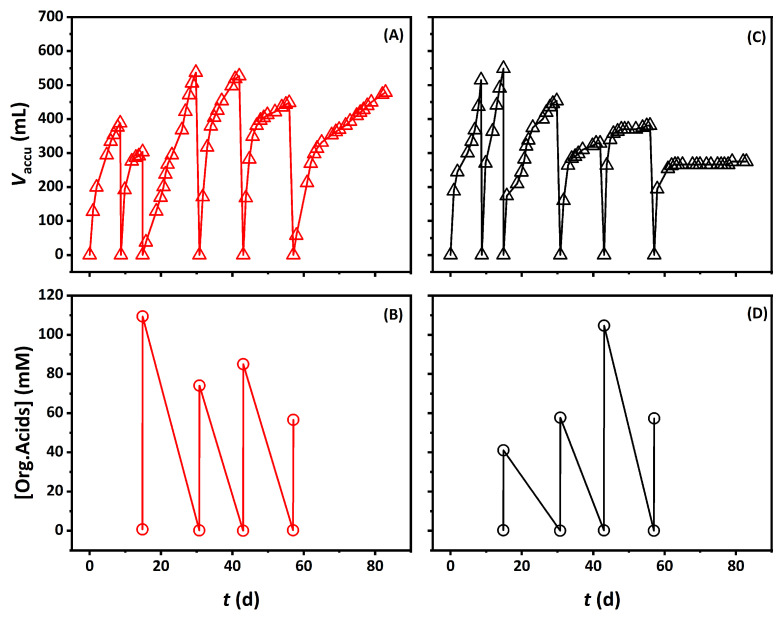
(**A**,**B**) Gas production and acetate degradation profiles of digestate sludge during the acclimation process; (**C**,**D**) Gas production and acetate degradation profiles of digestate sludge supplemented with carbon powder during the acclimation process. Open triangles: cumulative gas production for each cycle; open circles: acetate concentrations measured before and after each substrate replacement.

Digestate solids were acclimated for 82 days with five substrate replacements, resulting in six gas production cycles (Figure 8A). The first two replacement intervals were shorter (9 and 6 days), whereas the remaining intervals averaged 14 ± 2 days. The highest cumulative gas production reached 537.2 mL after the second substrate replacement, and the average cumulative gas production across all six cycles was 447 mL. Although the intended acetate concentration after each replacement was 54 mM, some measured values were substantially higher, likely due to inadequate mixing before sampling. Nevertheless, acetate was efficiently consumed within each cycle, decreasing to approximately 0.222 mM.

In the carbon powder-amended group (Figure 8C), digestate solids were acclimated under the same conditions for 82 days with five substrate replacements. The highest cumulative gas production reached 548.8 mL after the first substrate replacement, and the average cumulative gas production across the six cycles was 417 mL. Similar deviations between intended and measured acetate concentrations were observed, again likely due to incomplete mixing after substrate addition. However, acetate degradation was more complete in the carbon powder-amended group, with residual acetate decreasing to approximately 0.098 mM, indicating improved acetate utilization compared with the non-amended group.

### 3.4. Results of Power Generation Potential Tests for Acclimated Livestock Wastewater and Digestate Solids

#### 3.4.1. Power Generation Results of Raw Wastewater Solids After Acclimation

**Figure 9:**
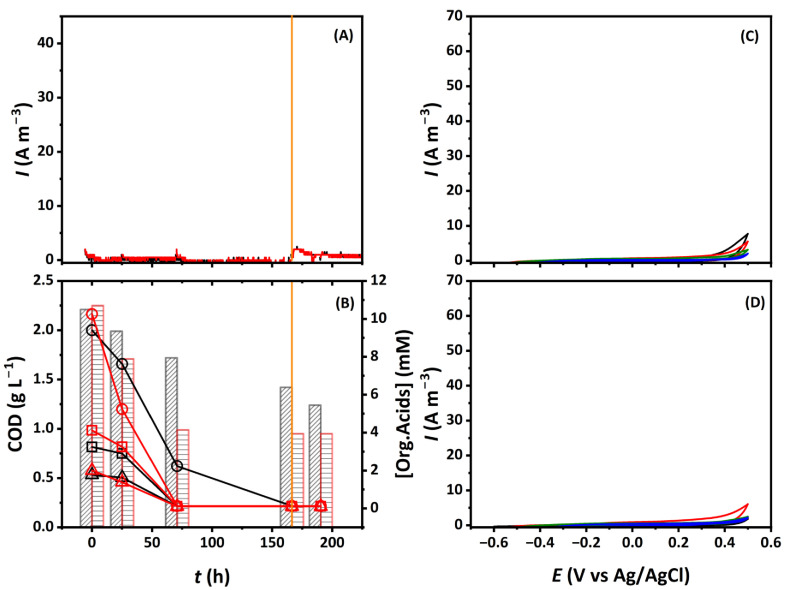
Profiles and cyclic voltammograms of wastewater solids acclimated for 54 days and cultured in electrochemical reactors over time. (**A**) Chronoamperometry (current vs. time); (**B**) COD degradation and organic acid degradation. Black: CH1 reactor data; Red: CH8 reactor data; Solid lines: current; Bars: COD concentration; Open circles: acetate concentration; Open squares: propionate concentration; Open triangles: butyrate concentration; Brown vertical line: time of potassium ferricyanide addition; (**C**) CV plots of reactor CH1; (**D**) CV plots of reactor CH8. Black: 0 d; Red: 1 d; Green: 3 d; Blue: 4 d.

In the first test, raw wastewater solids acclimated for 54 days were inoculated into reactors CH1 and CH8. During the 5-day operation, the current remained low (−1 to 2 A m^−3^), and no stable electricity generation was observed (Figure 9A). However, both reactors showed substantial COD removal, with efficiencies of 43.9% in CH1 and 57.8% in CH8 (Figure 9B). Acetate and butyrate were efficiently degraded in both reactors, whereas propionate removal was poor and unstable, particularly in CH8, where propionate accumulated over time. Addition of potassium ferricyanide at 167 h induced only a transient increase in current, indicating limited electron-transfer activity. CV analysis further showed that the initial redox signal at 0.4 V gradually disappeared. Together, these results indicate that although organic matter degradation occurred, the acclimated sludge did not establish effective electroactive performance.

#### 3.4.2. Power Generation Results of Digestate Sludge Acclimated with Carbon Powder

**Figure 10:**
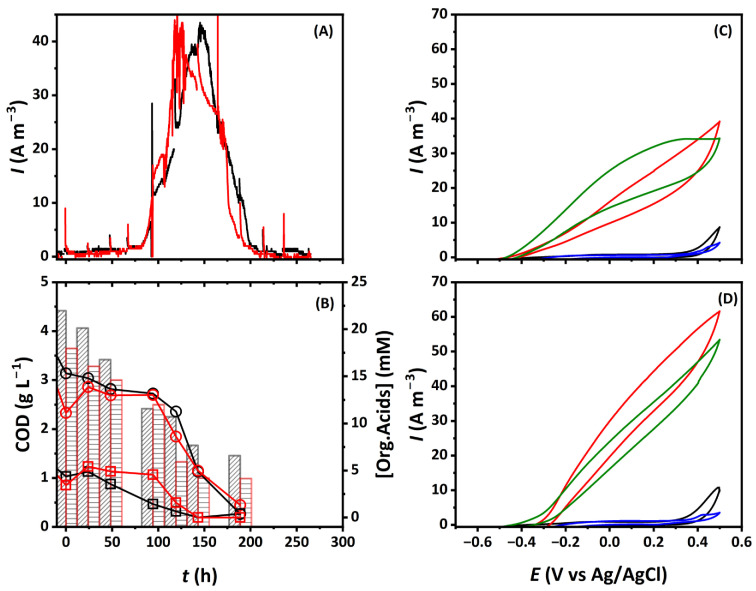
Cyclic voltammograms of digestate solids acclimated with carbon powder for 22 days and cultured in electrochemical reactors. (**A**) Chronoamperometry (current vs. time); (**B**) COD degradation and organic acid degradation. Black: CH1 reactor data; Red: CH8 reactor data; Solid lines: current; Bars: COD concentration; Open circles: acetate concentration; Open squares: propionate concentration; (**C**) CV plots of reactor CH1; (**D**) CV plots of reactor CH8. Black: 0 d; Red: 5 d; Green: 6 d; Blue: 11 d.

At *t* = 0, digestate sludge supplemented with carbon powder and acclimated for 22 days was inoculated into reactors CH1 and CH8. Over the 11-day experiment, both reactors showed substantial current generation with good reproducibility, reaching maximum currents of 8.5 mA in CH1 and 10 mA in CH8 (Figure 10A). Both reactors also showed marked COD degradation, with removal efficiencies of 67.0% in CH1 and 72.9% in CH8. Acetate and propionate were completely removed in both reactors, whereas butyrate was not detected. Because the current generation persisted after complete depletion of organic acids at 165 h, the acclimated microbial community was likely able to utilize other COD components present in the livestock wastewater.

CV measurements performed daily from day 0 to day 11 revealed a clear catalytic current response beginning around day 3. Both reactors showed maximum catalytic current on day 5, reaching approximately 35 and 55 A m^−3^ at +0.4 V in CH1 and CH8, respectively. The catalytic current remained detectable until day 10 and disappeared on day 11. A catalytic response was also observed from approximately −0.3 V, although the redox-active compounds responsible for this signal remain unknown.

#### 3.4.3. Power Generation Results of Acclimated Digestate Sludge

**Figure 11:**
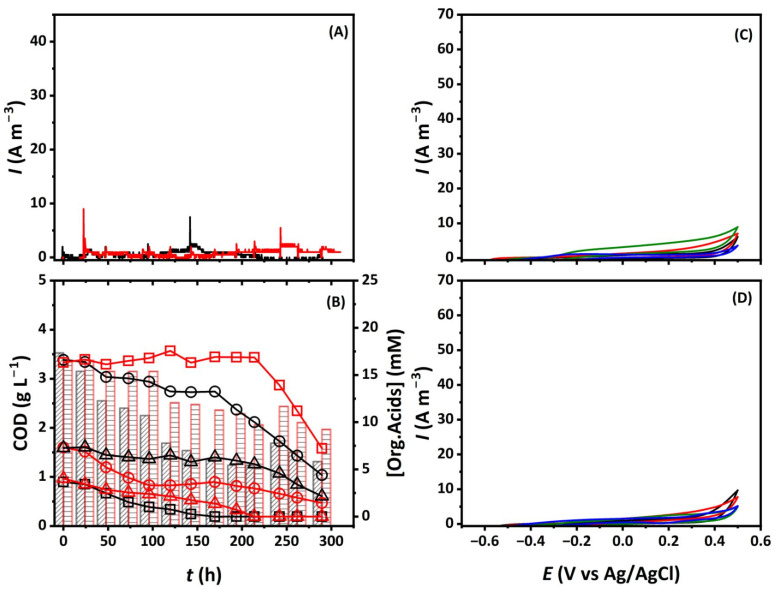
Cyclic voltammograms of digestate solids acclimated for 22 days and cultured in electrochemical reactors. (**A**) Chronoamperometry (current vs. time); (**B**) COD degradation and organic acid degradation. Black: CH1 reactor data; Red: CH8 reactor data; Solid lines: current; Bars: COD concentration; Open circles: acetate concentration; Open squares: propionate concentration; (**C**) CV plots of reactor CH1; (**D**) CV plots of reactor CH8. Black: 0 d; Red: 3 d; Green: 5 d; Blue: 11 d.

At *t* = 0, digestate sludge acclimated for 22 days without carbon powder was inoculated into reactors CH1 and CH8. During the 11-day experiment, both reactors showed only weak and delayed current generation, with transient increases to 0.4 mA observed after 140 h in CH1 and 243 h in CH8 (Figure 11A).

Compared with the carbon powder-acclimated digestate, COD and organic acid removal were less effective. By 290 h, both reactors showed only partial degradation of COD, acetate, and propionate. CV measurements further showed weaker current responses than those observed in the carbon powder-acclimated group, indicating that digestate sludge acclimated without carbon powder was less effective in establishing electron transfer pathways after inoculation into the electrochemical reactor.

### 3.5. Microbial Community Analyses

**Figure 12:**
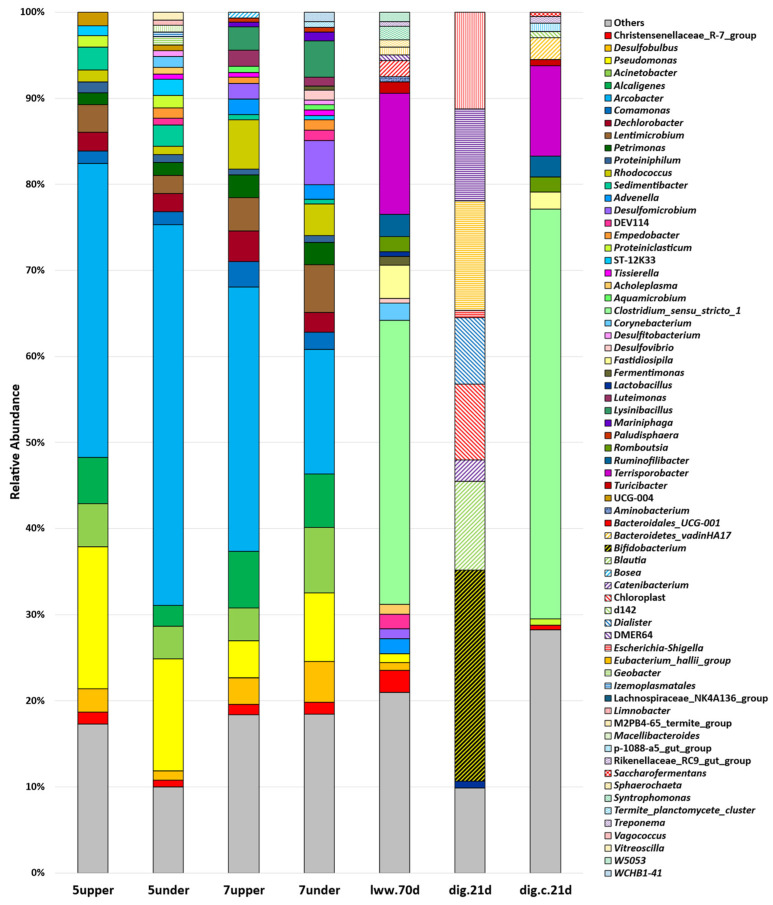
Genus-level microbial community composition of CH5 and CH7 reactor samples, acclimated raw livestock wastewater solids, and digestate-based enrichments determined by third-generation sequencing.

The genus-level microbial community structures of the different samples are shown in Figure 12. Clear differences in community composition were observed among the reactor samples, the acclimated raw livestock wastewater solids, and the digestate-based enrichments. The microbial communities in the CH5 and CH7 reactor samples exhibited relatively similar overall profiles. However, differences in the relative abundances of the dominant genera persisted between the upper and lower chamber samples. In contrast, the microbial community of the raw livestock wastewater solids after 70 days of acclimation (lww.70d) was clearly distinct from those of the reactor samples. This result is consistent with the poor electrochemical performance of the raw wastewater solids observed in the power generation tests.

The digestate-based samples also showed marked compositional differences. In particular, the microbial community of the carbon-powder-amended digestate after 21 days of acclimation (dig.c.21d) differed substantially from that of the digestate acclimated without carbon powder (dig.21d), indicating that carbon powder supplementation significantly altered the enrichment trajectory of the microbial community. Compared with dig.21d, dig.c.21d appeared to be dominated by fewer major genera, suggesting a stronger selective effect during acclimation. Overall, these results indicate that both reactor operation and carbon powder supplementation reshaped the microbial community structure, and the latter may have contributed to the improved electrochemical performance observed in the carbon-powder-acclimated digestate.

However, the microbial community analysis should be regarded as supportive evidence rather than direct mechanistic proof. Although the observed community shifts were consistent with the electrochemical results, no direct causal link was established between specific taxa and extracellular electron transfer activity. Therefore, the community data mainly support the conclusion that different inoculum sources and acclimation strategies led to distinct enrichment outcomes.

## 4. Discussion

This study demonstrates that liter-scale MFCs inoculated with acclimated sludge can simultaneously achieve electricity generation and COD removal from livestock wastewater. The design of the present liter-scale MFC reactor was adapted with reference to the long-term continuous-flow system reported by Lu et al. [20]. Their study demonstrated that this reactor configuration was well-suited for continuous operation, providing useful guidance for the engineering design of larger-scale MFC systems. In particular, the use of heat-treated cathode materials was considered an attractive feature for practical application, because such treatment can improve cathodic oxygen reduction reaction performance without requiring expensive catalysts [21,22,23]. From an engineering perspective, this design offers a reasonable balance between electrochemical performance, structural simplicity, and applicability to continuous wastewater treatment systems. The results also indicate that digestate solids from the Southern Taiwan livestock farm’s anaerobic digester contained microorganisms with electroactive potential, although proper acclimation was required before efficient current generation could be established. Reactor stability was one of the major limitations of this study. Although high COD removal was achieved during much of the operation, temporary decreases in treatment performance were observed in association with leakage, maintenance, and operational transition. Therefore, the present results demonstrate the feasibility of the liter-scale MFC system but are insufficient to support claims of stable long-term engineering operation [24,25]. It should also be noted that microbial deactivation or poisoning was not directly investigated in this study. Therefore, the temporary declines in performance observed during some operating periods cannot be conclusively attributed to these mechanisms.

Among the acclimation strategies tested, carbon powder addition clearly improved subsequent electrochemical performance. Compared with sludge acclimated without carbon powder, the carbon powder-amended sludge showed faster startup, stronger CV responses, and more effective COD and organic acid degradation after inoculation into the electrochemical reactors. These findings suggest that conductive carbon materials may have facilitated the enrichment or activation of electrochemically active microorganisms [25,26].

The gas production behavior during acclimation further suggests that carbon powder altered microbial electron flow. This effect may be discussed in the context of direct interspecies electron transfer (DIET) [27], in which conductive materials can facilitate electron exchange more directly than diffusible carriers such as H_2_ or formate. In the present study, the faster startup, stronger current response, and lower residual acetate observed in the carbon-powder-amended group were consistent with this possibility. However, because conductive materials may also affect biofilm formation and microbial enrichment in mixed cultures, the present results are more conservatively interpreted as evidence that carbon powder promoted electrochemical enrichment, rather than as direct proof of DIET [28].

After transfer into the electrochemical reactor, the carbon powder-acclimated sludge generated current much more rapidly than the non-amended sludge, suggesting that conductive particles promoted the formation of more effective electron transfer pathways. This interpretation is consistent with previous studies reporting that carbon-assisted external acclimation can accelerate MFC startup [19]. Because SEM imaging was not performed in the present study, we do not claim direct observation of nanowires or other extracellular conductive structures [29,30]. Instead, we interpret the results more conservatively as evidence that conductive-material-assisted acclimation improved the electrochemical readiness of the sludge community.

Overall, the comparison between Section 3.4.2 and Section 3.4.3 suggests that carbon powder acclimation promoted faster establishment of electrochemical activity, resulting in faster current generation and more effective substrate degradation. Thus, carbon-assisted acclimation appears to be a promising strategy for improving MFC startup and treatment performance in livestock wastewater applications, although the underlying mechanism remains to be further verified.

## 5. Conclusions

This study demonstrated that liter-scale MFCs can simultaneously remove organic matter from livestock wastewater and recover electricity, confirming their potential as a supplementary technology for wastewater treatment and energy recovery. Although both CH5 and CH7 achieved substantial COD removal, differences in reactor stability and performance also highlighted the importance of reactor integrity and operational control in scale-up applications. Among the inocula tested, raw wastewater solids showed limited electrochemical activity after acclimation, whereas anaerobic digestate exhibited clear potential for enrichment of electroactive microorganisms. In particular, carbon-powder-assisted acclimation significantly improved startup, current generation, catalytic current response, and removal of COD and organic acids, indicating that conductive material supplementation is an effective strategy for promoting electroactive community development. Microbial community analysis further showed that both reactor operation and carbon powder supplementation altered the final community structure, supporting the view that carbon-assisted acclimation not only enhances performance but also reshapes microbial enrichment pathways. Overall, this study suggests that anaerobic digestate is a more suitable inoculum source than raw wastewater solids for electroactive enrichment, and that carbon powder is a promising additive for accelerating startup and improving electrochemical performance in livestock wastewater-fed MFC systems.

## Figures and Tables

**Table 1:** Summary of water quality analysis data from Southern Taiwan livestock farm.

		Raw Wastewater				
		10/4	1/23	3/20	4/24	5/22
COD (mg L^−1^)		16,436 (876)	20,999 (0)	15,272 (0)	15,130 (6629)	14,583 (0)
NH4+ (mg-N L^−1^)		1136 (132)	1741.6 (0)	1462.2 (41.9)	1373.4 (22.6)	1110 (67.6)
Organic acids (mM)	Acetate	39.2	111.9	76.6	51.3	78.14
	Propionate	14.0	32.6	27.6	0	23.07
	Butyrate	7.0	18.8	15.7	0	10.72
TKN (mg-N L^−1^)		1210	2069 (600)	2548 (221.7)	2050 (35)	975 (153)
SS (mg L^−1^)		16,583 (833)	9833 (2592)	19,000 (333)	15,500 (500)	14,166 (236)
VSS (mg L^−1^)		13,750 (569)	7900 (1555)	16,000 (471)	12,666 (0)	11,333 (1414)

Values in parentheses represent standard deviations.

## Data Availability

The data, in parts or their entirety, is are available upon requests directed to the corresponding author.
